# Supplementary material for: The Effectiveness of a Foundation Year 1 Doctor Preparation Course for Final Year Medical Students
Source: J Med Educ Curric Dev. 2021 Jan 7;8:2382120520984184. doi: 10.1177/2382120520984184 (PMC7797572; doi:10.1177/2382120520984184)
Supplement: sj-pdf-1-mde-10.1177_2382120520984184 – Supplemental material for The Effectiveness of a Foundation Year 1 Doctor Preparation Course for Final Year Medical Students [file sj-pdf-1-mde-10.1177_2382120520984184.pdf]

Preparation 2 Practice Course – Course Programme

March 2015

Partnership Learning Centre, Good Hope Hospital

DAY 1				
8.30 – 8.45	Registration, Coffee and Biscuits			
8.45 - 9.00	Welcome and Introductions			
9.00 – 9.15	Introductory lecture<br>- Learning objectives, Content, Structure of the two days			
9:15 – 10:00	The UK Foundation Programme – portfolio, WBAs, CV tips, The next step, courses			
10:00 – 10:45	Lecture - Acute coronary syndrome and management			
10:45 – 11:15	Team A – Px for ACS + LVF	Team B – Px for ACS + LVF	Team C – Px for ACS + LVF	Team D – Px for ACS + LVF
11:15 – 11:45	Coffee and Biscuits			
11:45 – 12:15	Lecture – AF and common tachyarrhythmias			
12:15 – 12:45	Team A – Px for AF/Arrhythmias	Team B – Px for AF/Arrhythmias	Team C – Px AF/Arrhythmias	Team D – Px AF/Arrhythmias
12:45 - 13:30	Lunch			
13:30 – 14:15	Lecture – Approach to the septic patient			
14:15 – 15:00	Lecture – Approach to venous thromboembolism			
15:00 – 15:15	Coffee and Biscuits			
15:00 – 17:15	Small Group Sessions (What they don't teach you)			
	How to confirm death and death certificates	How to assess a patient after fall	Documentation	GI Bleed
15:15 – 15:40	Team A	Team B	Team C	Team D
15:40 – 16:05	Team D	Team A	Team B	Team C
16:05 – 16:30	Team C	Team D	Team A	Team B
16:30 – 16:55	Team B	Team C	Team D	Team D
:00 – 17:30	End of Day and Fun Quiz using Turning Point			

DAY 2				
8.30 – 8.45	Coffee			
8.45 - 9.00	Introduction to day 2			
9.00 – 9.45	Lecture – Approach to oliguric patient and managing acute renal failure			
9:45 – 10:00	Coffee and Biscuits			
10:00 – 12:00	Small Group Sessions (Prescribing)			
	Fluid management lecture	Prescribing Fluids	Prescribing sedation	Anticoagulation
10:00 – 10:25	Team A	Team B	Team C	Team D
10:25 – 10:50	Team D	Team A	Team B	Team C
10:50 – 11:15	Team C	Team D	Team A	Team B
11:15 – 11:40	Team B	Team C	Team D	Team D
12:00 – 13:00	Lunch			
13:00 – 15:00	Small Group Sessions (Survival Guide)			
	Radiology Revision	On-calls and night shifts	Handovers and Referrals	Abnormal blood results / ABGs
13:00 – 13:25	Team A	Team B	Team C	Team D
13:25 – 13:50	Team D	Team A	Team B	Team C
13:50 – 14:15	Team C	Team D	Team A	Team B
14:15 – 14:40	Team B	Team C	Team D	Team D
15:00 – 15:15	Coffee and Biscuits			
15:15 – 16:00	Lecture – Abnormal electrolytes			
16:00 – 16:45	Lecture – Approach to the patient in shock			
16:45 – 17:00	Conclusion, Feedback, Return of cheques			

Appendix 1: Course programme.
